# Supplementary material for: Mouse-to-human modeling of microglia single-nuclei transcriptomics identifies immune signaling pathways and potential therapeutic candidates associated with Alzheimer’s disease
Source: bioRxiv. 2025 Feb 8:2025.02.07.637100. Preprint. [Version 1] doi: 10.1101/2025.02.07.637100 (PMC11839086; doi:10.1101/2025.02.07.637100)
Supplement: 7 [file NIHPP2025.02.07.637100v1-supplement-7.pdf]

bioRxiv preprint doi: https://doi.org/10.1101/2025.02.07.637100; this version posted February 8, 2025. The copyright holder for this preprint (which was not certified by peer review) is the author/funder, who has granted bioRxiv a license to display the preprint in perpetuity. It is made available under aCC-BY 4.0 International license.

SUPPORTING INFORMATION

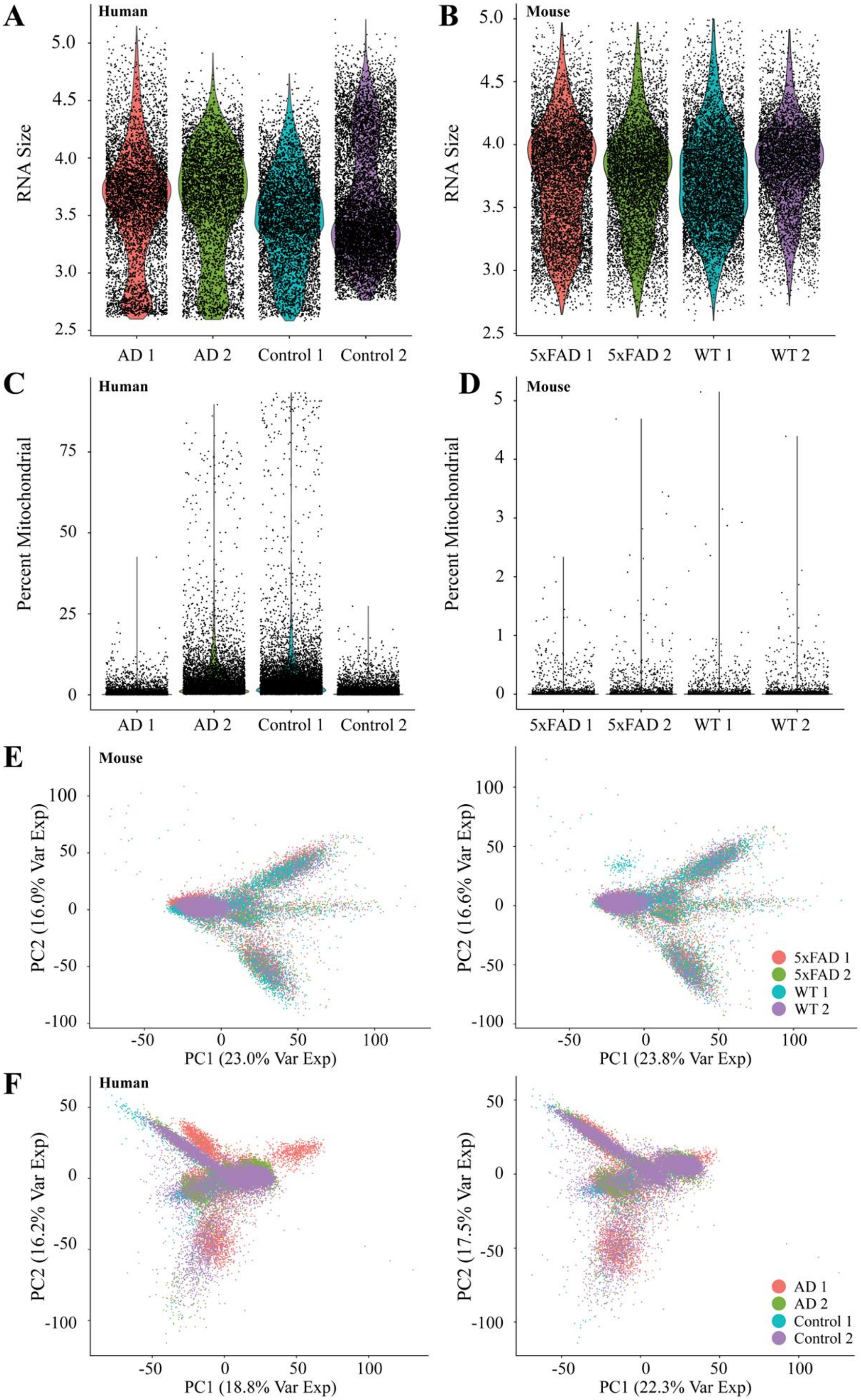

bioRxiv preprint doi: https://doi.org/10.1101/2025.02.07.637100; this version posted February 8, 2025. The copyright holder for this preprint (which was not certified by peer review) is the author/funder, who has granted bioRxiv a license to display the preprint in perpetuity. It is made available under aCC-BY 4.0 International license.

Supplementary Figure S1. Quality control of human and mouse single nuclei RNA-sequencing data. (A) Gene counts of human samples before QC filtering **(B)** Mitochondrial gene content of human samples before QC filtering **(C)** Gene count of mouse samples **(D)** Mitochondrial gene content of mouse samples **(E)** PCA plot of top two principal components in humans before harmony batch correction (left) and after harmony batch correction (right) **(F)** PCA plot of the top 2 principal components in mice before harmony batch correction (left) and after harmony batch correction (right)

bioRxiv preprint doi: https://doi.org/10.1101/2025.02.07.637100; this version posted February 8, 2025. The copyright holder for this preprint (which was not certified by peer review) is the author/funder, who has granted bioRxiv a license to display the preprint in perpetuity. It is made available under aCC-BY 4.0 International license.

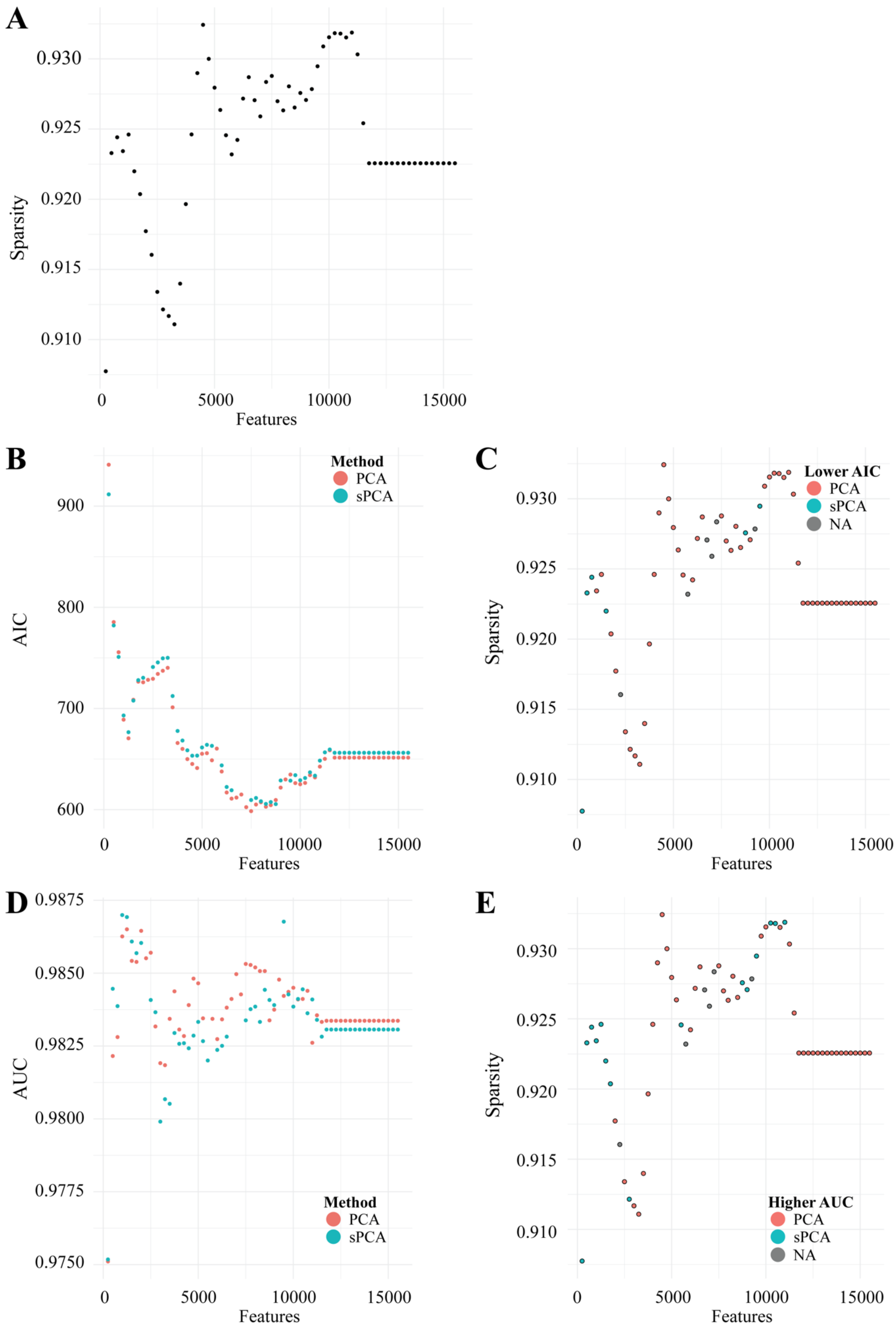

889

bioRxiv preprint doi: https://doi.org/10.1101/2025.02.07.637100; this version posted February 8, 2025. The copyright holder for this preprint (which was not certified by peer review) is the author/funder, who has granted bioRxiv a license to display the preprint in perpetuity. It is made available under aCC-BY 4.0 International license.

Supplementary Figure S2. (A) Sparsity of data frame as a function of variable features selected **(B)** AIC score as achieved through AIC minimization for PCA and sPCA as a function of variable features **(C)** Sparsity of data frame a function of variable features selected colored by lowest AIC **(D)** AUC score achieved through the ability of AIC minimized PCs to predict disease status as a function of variable features **(E)** Sparsity of data frame as a function of variable features selected colored by highest AUC
